# Investigating the Epidemiology and Socioecological Dynamics of Hydatid-like Cysts Within a Specific Endemic District

**DOI:** 10.3390/ani15111617

**Published:** 2025-05-30

**Authors:** Muhammad Irfan Khan, Rahmat Ali, Dejun Ji, Wei Lan, Ahmed A. Saleh, Shahab Ur Rehman, Muhammad Shuaib, Shah Zeb Ahmad, Hussain, Xi Wu

**Affiliations:** 1 College of Animal Science and Technology, Yangzhou University, Yangzhou 225009, China; mh23052@stu.yzu.edu.cn (M.I.K.); rahmatalihu@gmail.com (R.A.); rifatarro@gmail.com (W.L.); shoaibwzr@gmail.com (M.S.); shahzebahmad45@yahoo.com (S.Z.A.); 17712698357@163.com (X.W.); 2 Animal and Fish Production Department, Faculty of Agriculture (*Al-Shatby*), Alexandria University, Alexandria City 11865, Egypt; 3 Arid Zone Small Ruminants Research Institute, Ghulam Banda Kohat, Kohat 26010, Pakistan; 4 Department of Biotechnology and Genetic Engineering, Hazara University Mansehra, Dhodial 21300, Pakistan; hunny_khan38@yahoo.com

**Keywords:** echinococcosis, zoonotic diseases, parasite transmission, environmental influences, animal hosts, infection prevalence

## Abstract

Cystic Echinococcosis (CE) is a significant zoonotic parasitic disease caused by *Echinococcus* cestode parasites, affecting a variety of animals, including humans and wildlife, with serious implications for public health. In Pakistan, CE continues to pose a considerable public health challenge, impacting the livestock sector and wildlife conservation due to its economic and socio-cultural ramifications. This study aimed to examine the frequency of lesions indicative of *Echinococcus* cysts in livestock across several slaughterhouses in Khyber Pakhtunkhwa (KP), with the data collection occurring from May 2022 to February 2024. A total of 1120 animals, including 455 buffaloes, 295 cows, 200 sheep, and 170 goats, were assessed. The overall CE prevalence was found to be 5.7% (95% CI: 4.3–7.0%; 63 out of 1120), with the highest frequency in buffaloes (6.6%; 95% CI: 4.3–8.9%) and the lowest in goats (2.9%; 95% CI: 0.4–5.5%). Female animals demonstrated a higher infection rate (6.3%; 95% CI: 4.4–8.1%) compared to males (4.7%; 95% CI: 2.8–6.7%), with the liver being the most frequently affected organ (55.0%; 95% CI: 41.7–66.3%). Additionally, a survey of 200 respondents revealed that 74.5% of farmers were unaware of CE, and only 14.5% were taking preventive measures against parasites. These findings suggest a potential decline in the prevalence of this parasitic disease, likely due to advancements in veterinary services and modern farming practices.

## Simple Summary

Cystic Echinococcosis (CE) is a significant zoonotic disease caused by *Echinococcus* parasites, impacting both animals and humans while posing substantial public health and economic challenges in Pakistan. A study conducted between May 2022 and February 2024 analyzed 1120 livestock in Khyber Pakhtunkhwa, revealing an overall CE prevalence of 5.65%. Among the livestock examined, buffaloes had the highest infection prevalence at 6.59%, while goats had the lowest at 2.94%. Females exhibited a higher infection prevalence (6.25%) compared to males (4.74%), with the liver being the most commonly affected organ (54.96%). A survey indicated that 74.5% of farmers were unaware of CE, and only 14.5% implemented preventive measures. The findings suggest a potential gradual decline in CE prevalence, likely attributable to improvements in veterinary care and modern farming practices.

## 1. Introduction

Echinococcosis is a zoonotic disease caused by cestode parasites, predominantly manifesting in two main forms: Cystic Echinococcosis (CE) and Alveolar Echinococcosis (AE) [1]. AE is caused by *Echinococcus multilocularis*; CE is associated with *Echinococcus granulosus sensu lato* [2,3]. CE represents the more widespread form of this disease, accounting for approximately 80% of documented cases [4,5] and significantly impacting regions globally, particularly those with extensive livestock farming. Regions globally, especially those with extensive livestock farming, are significantly impacted by CE. This parasitic disease reveals a strong correlation with areas where hosts such as sheep, goats, cattle, and camels play a crucial role as intermediate hosts in the pathogen’s life cycle. The geographical distribution of this zoonotic disease aligns closely with regions characterized by substantial populations of these domesticated animal species [6].

The adult form of *E. granulosus*, classified under the order Cyclophyllidea, resides in the small intestines of definitive hosts, primarily canines. While the tapeworm primarily affects canines, it poses significant health threats to intermediate hosts (e.g., sheep, cattle) and accidental dead-end hosts, including humans, leading to CE [7]. *E. granulosus* affects both humans and a variety of domestic animals [8]. Humans can unintentionally become intermediate hosts for the parasite by consuming food or water contaminated with *Echinococcus* eggs, which are shed by definitive hosts, such as canines [9].

In intermediate hosts like sheep, goats, buffaloes, horses, pigs, and humans, the parasite can develop cysts of varying sizes, comparable to a ping-pong ball in sheep, a tennis ball in horses, and as large as a football in other species. This condition, referred to as hydatid disease or CE, results from the invasion of the oncosphere (embryo) [10]. *Echinococcosis* exhibits a high incidence among livestock and canine populations in certain regions, such as parts of South America, the Middle East, and Asia, resulting in significant economic ramifications due to the condemnation of affected meat products. CE is particularly prevalent in regions where herding is common, with a global distribution encompassing northern Ethiopia, Sudan, northern Iran, and Pakistan, countries all suffering major losses in livestock [11,12,13,14]. Reports from Kazakhstan indicate a high prevalence in cattle, sheep, and herding dogs, underscoring the urgent need for effective control measures [15]. The World Health Organization (WHO) recognizes hydatid disease as a tropical ailment that has long been undervalued, resulting in substantial adverse effects on the health of both human and animal populations. In regions where it is endemic, this condition leads to significant morbidity and mortality in livestock, culminating in considerable economic losses [16,17]. Research on its frequency and molecular identification is crucial for developing effective control strategies and preventive measures to mitigate the economic impact of CE [18].

In Pakistan, the livestock industry forms the backbone of agricultural activities. Over 8 million rural households depend on agriculture, with livestock production contributing 35–40% of their income [19]. The livestock industry is vital to Pakistan’s economic stability, contributing approximately 11.9% to the national gross domestic product (GDP), while agriculture accounts for about 21%. According to the Economic Survey (2019–2020), the livestock distribution in Pakistan includes approximately 31.2 million ovines, 78.2 million caprines, 49.6 million bovines, and 41.2 million water buffaloes, making it a critical component of the agricultural economy, with a total meat production of nearly 4708 tons [19]. Nevertheless, CE has severely impacted Pakistan’s livestock sector, resulting in substantial economic losses [20,21]. Parasitic infections are estimated to cause an annual economic loss of PKR 26.5 million, with specific losses attributed to *E. granulosus* assessed at USD 276.20 per 100 sheep or goats and USD 165.72 per 100 cows, camels, or buffaloes [22,23,24].

Research on the prevalence of *E. granulosus* in Pakistan is limited, with inadequate data concerning its geographical distribution and the causative agents of CE [21], particularly in the Malakand District of Khyber Pakhtunkhwa (KP) [25]. Despite these limitations, existing studies provide some understanding of the issue. The infection prevalence in livestock across various regions of Pakistan ranges from 2.4% to 35% [8], with studies in Punjab reporting proportions of 6.22% in cattle and buffalo, and 6.67% across a broader range of livestock, including sheep and goats [23]. Recent molecular studies have advanced this understanding; for instance, Alvi et al. [22] employed complete mitochondrial *cox1* and *nad1* gene sequencing to identify the dominant G1 (sheep) and G3 (buffalo) strains in Punjab and Sindh provinces, while also reporting the first detection of the G5 genotype (*E. ortleppi*) in Pakistan [23]. Their findings revealed high haplotype diversity and signals of recent population expansion, suggesting dynamic transmission patterns. Despite these insights, gaps persist in mapping the geographical distribution of CE strains, particularly in the Malakand District of Khyber Pakhtunkhwa (KP) [25]. Prevalence rates in livestock across Pakistan range from 2.4% to 35% [8], with regional variations, such as 6.22% in Punjab cattle and buffalo [23] and 2.4–17.4% in KP livestock [24]. The predominance of G3 in buffalo aligns with findings in neighboring India, underscoring cross-border transmission risks. These molecular and epidemiological disparities highlight the need for integrated surveillance to inform region-specific control strategies.

This study aims to investigate the epidemiology of CE in domestic animals (cows, buffaloes, goats, and sheep) within the Malakand District of Khyber Pakhtunkhwa (KP), Pakistan, focusing on five sub-regions: Batkhela, Bajawar, Chakdara, Dargai, and Thana. The research objectives include identifying the preferred organs for cyst formation and assessing the viability of protosciences. A questionnaire survey was conducted to gather demographic data and identify the risk factors associated with CE, focusing on livestock and dog ownership, management practices, and farmers’ awareness of the disease. By analyzing the responses, we can identify potential hotspots for disease emergence based on factors such as livestock density, farming practices, and community awareness. This information is crucial for targeted interventions and understanding the socioecological dynamics contributing to the transmission of CE in the region. Additionally, gauging the community’s awareness of CE and prevention strategies is essential for establishing an effective control program. Consequently, this study evaluates CE awareness among livestock farmers across various provincial regions, which is significant for addressing the spread of infection.

## 2. Materials and Methods

### 2.1. Study Area

The present research was conducted in the Malakand District of KP, Pakistan, encompassing five sub-regions: Batkhela, Bajawar, Chakdara, Dargai, and Thana. The data collection spanned from May 2022 to February 2024, with slaughterhouse inspections and farmer surveys conducted across these localities (Supplementary File S1, Figure S1). During the study period, a total of 1120 animals, which included buffaloes, cows, goats, and sheep, were examined, revealing CE in 63 animals. This study involved the examination of hydatid cysts collected from the organs of slaughtered livestock in the aforementioned district of KP, Pakistan.

The province of KP, situated in the northwestern region of Pakistan, is one of the country’s four administrative provinces. Despite being the smallest province in terms of land area, KP ranks as the third-largest province by population and economic significance. This study focuses on the frequency of CE in domestic livestock across five sub-regions (Figure S1).

### 2.2. Study Design

The present research, conducted from May 2022 to February 2024, involved the examination of hydatid cysts collected from the organs of slaughtered livestock in the aforementioned district of KP, Pakistan.

### 2.3. Antemortem Examination

Samples were collected from ten slaughterhouses across the district of Malakand in KP. The study included examinations of cattle at the same abattoirs during regular visits conducted three days per week throughout the year. Among the 1120 slaughtered animals, 85.7% (960/1120) were sourced from various marketplaces in and around the local area, while the remaining 14.3% (160/1120) came directly from local farms.

A comprehensive record was compiled that encompassed cyst data as well as detailed information about the animals, including the localization of cysts within visceral organs, age, and sex. Age determination was initially conducted through inquiries with intermediaries (buyers) and was further corroborated through assessments of dental eruption and oral examinations [26]. The slaughtered animals were categorized into three age groups: Group 1 included animals ranging from 1 to <3 years, Group 2 comprised animals aged 3 to 5 years, and Group 3 consisted of animals over 5 years of age.

### 2.4. Abattoir Survey and Post-Mortem Examination

This study examined a total of 1120 animals between May 2022 and February 2024 (Table 1). This investigation included 455 buffaloes, 295 cows, 200 sheep, and 170 goats, representing various age groups across these species. Following the slaughter process, a comprehensive post-mortem evaluation was conducted, which included a visual assessment and a tactile examination of the visceral organs.

The liver, lungs, spleen, kidneys, and heart were extracted from the slaughtered animals and meticulously examined for cystic formations. Following extraction, each organ (liver, lungs, spleen, kidneys, heart) was systematically dissected to expose internal structures, enabling the thorough inspection of both superficial and embedded cysts. The cysts were counted manually, and their dimensions (length/width) were measured using calipers. The infection severity was categorized as follows: (a) Minor: 1–5 cysts occupying ≤25% of the organ’s volume. (b) Intermediate: 6–15 cysts occupying 26–50% of the organ’s volume. (c) Major: >15 cysts or >50% organ involvement. This classification was validated against gross pathology standards [27].

### 2.5. Questionnaire Survey

To gather demographic and relevant data regarding Cystic Echinococcosis (CE) transmission risks, a comprehensive questionnaire study was developed. The survey was administered orally in the primary indigenous languages of the research locale, specifically Pashto and Urdu, to ensure effective communication with participants.

The questionnaire was developed through a structured process involving the following: (a) A literature review: a comprehensive analysis of Echinococcosis and zoonotic diseases to identify key variables (demographic, social, ecological, and epidemiological factors). (b) Expert consultation: collaboration with specialists in veterinary science, epidemiology, and local farming practices to refine questions for cultural relevance, alignment with study objectives, and validity enhancement.

Following the expert consultations, a pilot test of the questionnaire was conducted with a randomly selected group of farmers (n = 20) from the study area to assess the clarity and relevance of each question, as well as the response options. Modifications were made based on feedback regarding any ambiguous phrasing or logistical challenges encountered during the interviews. The reliability of the questionnaire was further supported by a Cronbach alpha value of 0.79, indicating that the instrument is reliable for assessing the risk factors associated with CE.

After the pilot testing, the questionnaire was finalized, and a content validation check was conducted by the same experts involved earlier, ensuring that the questionnaire accurately measured the intended constructs.

For the main survey, households were selected randomly. A stratified random sampling approach was employed to select participants. First, a comprehensive registry of livestock owners was compiled in collaboration with local agricultural offices, encompassing all households in the Malakand District. The registry was stratified into four subgroups based on the primary livestock type (buffaloes, cows, sheep, goats). Within each stratum, the participants were randomly selected using a computer-generated random number sequence (Microsoft Excel, RAND function). This ensured the proportional representation of each livestock category and an equal inclusion probability for all farmers. The final sample comprised 200 farmers, with 50 participants allocated to each stratum to maintain balance. The farmers who declined participation were replaced by reselecting from the same stratum to preserve the study’s demographic integrity. This registry was developed in collaboration with local agricultural extension offices and community leaders to promote inclusivity. The focus was on households owning at least one livestock animal or dog, essential for understanding the transmission dynamics of Echinococcosis. Participation was entirely voluntary, with informed consent obtained from each respondent prior to beginning the survey. The participants were made aware of the study’s purpose, the use of the information provided, and their right to withdraw at any time without consequence.

The questionnaire was designed with simple, close-ended questions to facilitate understanding and response accuracy (Supplementary File S2, Tables S1–S3). Options were formatted to allow the respondents to circle their answers, enhancing accessibility and precision in data collection. The data collected encompassed the population structure alongside social, ecological, and epidemiological factors influencing the transmission and persistence of *Echinococcosis* [28]. Key themes from Table S3 were integrated into the questionnaire to assess farmers’ awareness, dog ownership practices, and behaviors impacting the CE transmission risk. For instance, specific questions addressed the awareness of how CE affects animal organs, the potential for transmission to humans, the role of dogs as hosts for the parasite, and practices related to dog care and livestock management.

The survey design encompassed questions on farmers’ knowledge, such as whether they knew how CE impacts animal organs like the liver and lungs and whether they recognized that dogs can transmit diseases to livestock. This comprehensive approach aimed to deepen the understanding of the factors contributing to the prevalence of this zoonotic disease within the local context. The complete version of the questionnaire, reflecting these critical insights, can be found in the Supplementary Materials, ensuring the transparency and reproducibility of the study.

### 2.6. Statistical Analysis

A statistical analysis was conducted using SASS, 2012. Chi-square tests were employed to evaluate associations between categorical variables, ensuring the analysis was suitable for the nature of the data. For instance, with small sample sizes, Fisher’s Exact Test was utilized to maintain the validity of our findings. All the statistical tests were conducted at a significance level of 0.05, with adjustments for potential confounders applied as needed.

The questionnaire data were analyzed using SPSS version 25. To ensure adequate representation, the demographic profiles of the participants were aligned with the latest district census data. The participants’ knowledge regarding Cystic Echinococcosis (CE) was quantified through their correct responses to 20 targeted questions, reflecting their understanding of relevant transmission risks. The total knowledge scores were computed, assuming an equal contribution from each question to the overall assessment.

## 3. Results

Over a period of 22 months (from May 2022 to February 2024), animals were examined across various slaughterhouses. This cohort included 455 buffaloes, 295 cows, 200 sheep, and 170 goats.

Samples were collected from ten slaughterhouses located in five sub-regions of Malakand District: Batkhela (2 slaughterhouses), Thana (2), Dargai (2), Bajawar (2), and Chakdara (2). The number of animals examined per slaughterhouse and species is detailed in Table S4. For instance, the Batkhela slaughterhouses collectively processed 220 animals (buffaloes: 90; cows: 60; sheep: 40; goats: 30), while the Chakdara slaughterhouses examined 200 animals (buffaloes: 80; cows: 50; sheep: 40; goats: 30).

The overall infection rate exhibited a significant reduction of 5.62% across all the species (*p* < 0.05), with a 95% confidence interval of [4.48%, 6.76%]. Buffaloes demonstrated the highest frequency, at 6.59% (30 out of 455; 95% CI: [4.50%, 9.52%]), followed closely by cows, at 5.76% (17 out of 295; 95% CI: [3.31%, 9.25%]), and sheep, at 5.5% (11 out of 200; 95% CI: [2.75%, 10.35%]), while goats recorded the lowest proportion, at 2.94% (5 out of 170; 95% CI: [0.69%, 10.27%]). Detailed results regarding the overall frequency of hydatid cyst infections among the slaughtered animals are presented in Table 1.

### 3.1. Region-Wise Frequency of Cystic Echinococcosis in Buffaloes, Cows, Sheep, and Goats

The analysis of CE frequency in animals by region indicated that the highest infection rates were found in the sub-regions of Bajawar and Thana, which had rates of 6% (18 out of 300; 95% CI: [4.32%, 8.24%]). This was closely followed by Dargai at 5.5% (11 out of 200; 95% CI: [2.19%, 10.29%]) and Batkhela at 5.45% (12 out of 220; 95% CI: [2.28%, 10.92%]). The lowest frequency was recorded in Chakdara at 5% (10 out of 200; 95% CI: [2.02%, 9.88%]) (Table 2).

### 3.2. Organ-Wise Frequency of Cystic Echinococcosis in Buffaloes, Cows, Goats, and Sheep

**Figure 1:**
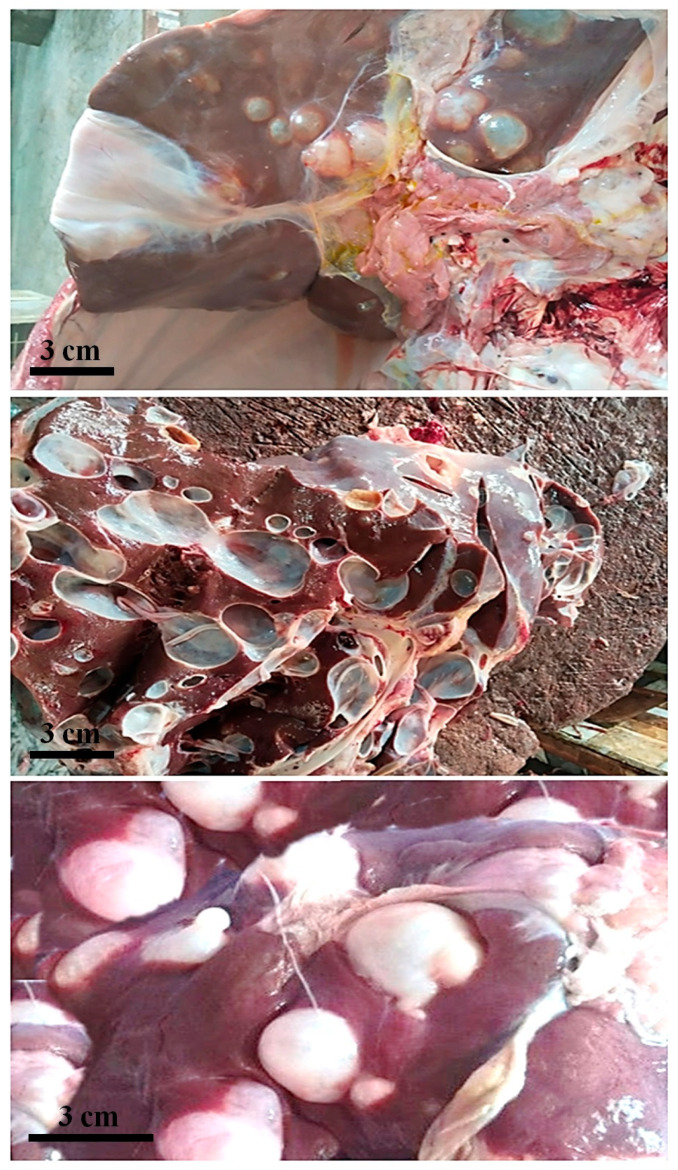
Appearance of hydatid cysts (HCs) in liver and lungs of slaughtered livestock from Khyber Pakhtunkhwa, Pakistan. Photographs depict cysts in buffaloes, cows, sheep, and goats. All images are original to authors.

Among the 63 organs found to be positive for hydatid cysts (Figure 1), the liver was the most commonly affected, comprising 34 cases (53.9%; 95% CI: [43.8%, 63.8%]), followed by the lungs, with 25 cases (39.6%; 95% CI: [30.2%, 49.5%]); the kidneys accounted for 2 cases (3.17%; 95% CI: [0.5%, 11.6%]). No infections were found in spleens across the examined species. Out of a total of 30 buffaloes with CE-like lesions, 18 had cysts in their livers (60%; 95% CI: [43.2%, 75.1%]), 10 in their lungs (33.3%; 95% CI: [18.2%, 51.4%]), and 1 each in their kidneys and hearts (3.3%; 95% CI: [0.1%, 17.0%]). For cows, the infection rates were 52.9% (9 out of 17; 95% CI: [31.2%, 73.0%]) for liver cysts; 41.1% (7 out of 17; 95% CI: [21.7%, 63.1%]) for lung cysts; and 5.8% (1 out of 17; 95% CI: [0.1%, 30.0%]) for kidneys, with no infections in the hearts or spleens. Sheep exhibited infection rates of 45.45% (5 out of 11; 95% CI: [19.7%, 74.6%]) for liver cysts and 54.5% (6 out of 11; 95% CI: [30.4%, 77.1%]) for lung cysts. In contrast, goats showed infection rates of 40% (2 out of 5; 95% CI: [7.4%, 83.0%]) for both liver and lung cysts, and 20% (1 out of 5; 95% CI: [0.5%, 71.8%]) for heart cysts. A detailed organ-specific cyst distribution and severity classification are provided in Table S5. These findings highlight that the liver is the most susceptible organ to hydatid cyst infection across all examined livestock species, with buffaloes showing the highest infection rates, particularly in the liver (60%) (Table 3).

### 3.3. Age-Wise Distributions of Buffaloes, Cows, Goats, and Sheep

This study identified notable differences in the frequency of CE across various age groups in animals. The CE frequency was significantly higher in older animals compared to their younger counterparts. Specifically, animals aged 1 to 3 years exhibited the lowest susceptibility, at 3.42% (13 out of 380; 95% CI: [1.93%, 5.96%]), while those aged 3 to 5 years showed a frequency of 5.44% (16 out of 294; 95% CI: [3.45%, 8.02%]). In contrast, animals over 5 years of age had a much higher infection frequency of 7.62% (34 out of 446; 95% CI: [5.60%, 10.00%]) (Table 4). The observed higher prevalence of CE in older animals is likely attributable to cumulative environmental exposure over time, as prolonged contact with *E. granulosus* eggs increases the probability of infection, compounded by the slow growth of hydatid cysts, which may take years to become detectable.

### 3.4. Sex-Wise Distribution of Cattle

The animals were categorized into two distinct classifications based on biological sex: male and female. The analysis of the sex-specific distribution of the parasite revealed a higher frequency of CE in adult female livestock, at 6.25% (41 out of 656; 95% CI: [4.35%, 8.75%]), compared to 4.74% (22 out of 464; 95% CI: [2.77%, 7.90%]) in males (Table 5). While the frequency in females was higher, the difference was not statistically significant.

### 3.5. Questionnaire Survey

#### 3.5.1. Demographic Profile and Livestock Farming Practices of Respondents in the Surveyed Regions

A questionnaire survey was conducted to assess the relevance of respondents to the study, as well as their practices and approaches within the investigated region. A total of 200 respondents were distributed across five areas: Dargai (15.5%), Batkhela (32%), Thana (14.5%), Chakdara (20%), and Bajawar (18%). This regional distribution ensured a diverse representation of farmers from different locations within the study area.

In terms of sex, the majority of respondents were male (93.5%), while females constituted only 6.5% of the participants, reflecting the male-dominated nature of farming activities in the surveyed regions. The respondents’ ages ranged from 22 years to above 40 years, with a small proportion (13.5%) aged 22–28, 21% aged 29–34, 28.5% in the 35–40 age group, and the largest group (37%) comprising individuals above 40 years. Most of the respondents (73.5%) were the heads of their households, while the remaining 26.5% were dependents, indicating that the majority of the participants were decision-makers in their households.

Additionally, 68% of the respondents resided in rural areas, while 32% were from urban areas, highlighting the predominantly rural nature of farming in these regions. Only 15.5% of respondents were migrants or refugees, with the remaining 84.5% being native to their respective regions. The education levels among the respondents exhibited significant variation: approximately 32.5% had completed primary education, 17.5% had attained middle school education, 7.5% had completed matriculation (the final examination taken after secondary school) or higher, 6% had received religious education, and 36.5% were illiterate. This diversity in educational attainment underscores the urgent need for programs aimed at improving literacy in farming communities.

Livestock farming was the primary source of income for 45.5% of the respondents, while 34% relied on crop farming, and 20.5% reported other income sources. Experience in livestock farming also varied significantly among the respondents; a small proportion (6%) had 1–4 years of experience, 27% had 5–9 years, 39.5% had 10–14 years, 22% had 15–19 years, and 5.5% had over 20 years of experience. These findings highlight the considerable experience of many farmers in livestock husbandry, which can serve as a valuable resource for developing sustainable farming practices in the region (Table S1). Briefly, the majority of the respondents were above 40 years old (37%), with the lowest proportion (13.5%) belonging to the 22–28 age group (Table S2).

#### 3.5.2. Animal Housing and Feeding Practices

The majority of the respondents housed their animals inside their homes (66%), while 34% kept them outside. In terms of the species raised, buffaloes were the most common (39%), followed by cows (23%), sheep (18.5%), and goats (12%), with 7.5% of the respondents raising more than one species. Regarding the feeding frequency, most farmers fed their animals twice daily (60.5%), followed by those who fed them three times or more (33.5%), and only 6% fed their animals once per day. Natural grazing was the primary feed source for the animals (51%), while 24% relied on purchased feed, 14.5% used homemade feed mixes, and 10.5% utilized other types of feed (Table S2).

#### 3.5.3. Health and Water Management

Only 35.5% of the respondents provided supplements or vitamins for their animals, while 64.5% did not. Fresh water was provided daily by 56.5% of farmers, twice daily by 21.5%, and three times daily by 13.5%, while 9% provided water less frequently. Wells (66.5%) served as the primary source of water, followed by streams (14.5%) and rivers (6%). Regarding cleaning practices, 80.5% reported cleaning animal housing daily, 15% did so weekly, and 4.5% cleaned monthly (Table S2).

#### 3.5.4. Disease Management and Awareness

Most of the farmers (70.5%) monitored and managed animal pregnancies, while 19.5% did not, and 10% did so occasionally. Regular vaccination against common livestock diseases (e.g., foot-and-mouth disease and *hemorrhagic septicemia*) was reported by 46% of the respondents yearly, 25.5% quarterly, and 13% monthly, while 15.5% did not vaccinate their animals. The awareness of CE was low, with only 20.5% of the respondents aware of the disease; among them, 65.85% identified the *Echinococcus* parasite as its cause. However, only 14.5% of the farmers (29/200) reported implementing preventive measures against parasites, while 85.5% (171/200) did not adopt any control practices (Table S2).

#### 3.5.5. Dog Ownership and Management

A significant proportion of the farmers (85%) kept dogs on their farms, primarily for guarding livestock (56.8%), while 24.26% kept them for companionship and 11.8% for herding. Most dogs (66.5%) were allowed to roam freely with the livestock, and 94.67% had direct contact with animals daily. However, only 9.5% of the farmers reported deworming their dogs regularly, while 29.58% vaccinated dogs against common diseases (e.g., rabies and distemper). Despite this, 95% of the respondents were aware of measures to prevent disease transmission from dogs to livestock, primarily through proper waste disposal and maintaining separate living areas (Table S3).

#### 3.5.6. Slaughter and Offal Disposal Practices

In terms of slaughtering practices, 57% of the respondents reported slaughtering livestock at home in the past year. During Eid ul-Adha “a period marked by significant religious observance, many families prefer the convenience and tradition of home slaughtering to partake in the festival’s customs”, 81% conducted slaughtering at home, while only 9.5% used slaughterhouses. Most of the respondents (53%) disposed of offal in open areas, 21.5% discarded it into rivers, and 16.5% buried it (Table S3).

## 4. Discussion

CE represents a significant economic burden on the livestock sector due to its adverse effects on animal health, leading to morbidity and mortality in food-producing animals and necessitating the disposal of vital organs [29]. Therefore, it is imperative to gather reliable epidemiological data on CE to establish a benchmark for future research, which will be instrumental in developing effective control strategies. In this aspect, the epidemiological landscape of CE in Malakand District is shaped by ecological and socioecological dynamics. Mountainous terrain and environmental conditions favor the survival of Echinococcus eggs, while traditional free-range grazing practices increase livestock exposure to contaminated areas. Cultural reliance on livestock for economic stability promotes communal grazing, further heightening infection risks. Limited access to veterinary services and financial constraints hinder preventive measures such as deworming, exacerbating transmission. These factors collectively underscore the need for integrated control strategies addressing both the environmental and behavioral drivers of CE.

This study reveals insights into the CE infection frequency among various species in the region, addressing a gap in the existing literature concerning the CE incidence in livestock [23,29,30,31,32,33,34,35,36,37,38,39,40,41,42,43,44,45,46,47,48] in Pakistan [21,23,27,30,31,32,33,34,35,36]. Infection rates vary globally, with reported rates of 0.58% in India [37], 10.6% in Italy [38], and 31.4% in Ethiopia [39].

The observed CE prevalence of 5.62% in KP aligns with recent declines reported in Pakistan (6.22% in Punjab [48]) but remains lower than rates in Ethiopia (31.4% [39]) or Moldova (59.3% [45]). This regional variation may reflect differences in livestock management, climate, or veterinary interventions. For instance, Punjab’s prevalence decline to 6.22% [48] mirrors our findings, likely due to improved veterinary services and modern farming practices. However, KP’s prevalence is higher than in India (0.58% [37]), where stricter abattoir regulations and community awareness programs are implemented [49,50,51].

The liver was the most frequently affected organ (54.96%), consistent with global trends where hepatic cysts dominate due to portal circulation filtering oncospheres [6,10,52,53,54,55]. Similar findings were reported in Punjab, Pakistan (liver: 58% [22]), Ethiopia (63% [55]), and Iran (67% [53]). However, the absence of splenic cysts contrasts with studies from Ethiopia [55] and Iran [53], where splenic involvement reached 5–8%, possibly due to regional differences in parasite strain tropism or host immunity [8].

Older animals (>5 years) exhibited the highest infection rates (7.62%), corroborating studies in Ethiopia [39] and Kazakhstan [15], where prolonged exposure to contaminated environments increases the risk. However, the age-related increase in CE prevalence (7.62% in animals > 5 years vs. 3.42% in 1–3-year-olds) likely reflects the cumulative exposure over time rather than an inherent biological susceptibility. Hydatid cysts grow slowly, requiring months to years to become detectable, which aligns with findings in Ethiopia [39] and Kazakhstan [15]. Thus, the higher prevalence in older animals may result from prolonged environmental exposure to *E. granulosus* eggs, rather than an elevated risk per se. This distinction is critical for designing control strategies targeting transmission pathways (e.g., deworming dogs, improving sanitation), rather than age-specific interventions. Females showed higher susceptibility (6.25% vs. 4.74% in males), aligning with hormonal or physiological factors proposed in sheep and cattle [31,43]. For example, pregnancy-induced immunosuppression in females may elevate susceptibility, as noted in Italian cattle [38].

Globally, the inadequate awareness of CE and the suboptimal adoption of preventive measures among livestock farmers remain critical barriers to disease control, as evidenced by studies across diverse socioecological settings [35,39,56,57,58,59,60,61,62,63,64,65]. The low farmer awareness of CE (74.5% unaware) mirrors findings in Morocco [56] and Tibet [65], where poor literacy and limited veterinary outreach perpetuate transmission risks. Only 14.5% implemented preventive measures, contrasting with Punjab, where 28% of farmers have adopted deworming [48]. This gap underscores the need for targeted education, as successful CE control in Tasmania [57] and Sardinia [64] relies on farmer training programs.

Unrestricted dog–livestock contact (94.08%) and poor deworming (9.5%) align with the high CE prevalence in pastoral communities globally [8,66]. In Moldova, unrestricted dog access correlated with 59.3% CE prevalence [45], while in Sardinia, controlled dog management reduced transmission [64]. Home slaughter (81%) and open offal disposal (53%) further exacerbate risks, echoing studies in Libya [63] and China [65], where such practices sustained parasite cycles.

The gradual decline in the CE prevalence (from 8.71% in 2018 [21] to 5.62% in this study) parallels trends in Punjab [48] and reflects improved veterinary care. However, challenges persist, as seen in Ethiopia, where CE remains endemic despite similar efforts [39]. A One Health approach integrating livestock deworming, dog vaccination, and community education is critical, as demonstrated in China [58] and Iran [59].

In Pakistan, regional variation exists, as indicated by various studies [23,31,34,42,43]. Our research indicated a moderate endemic level of hydatid infection in Malakand and Bajaur, with a frequency rate of 5.62%, suggesting a concerning yet manageable level of endemicity.

A contributing factor to the prevalence of CE is the lack of regulatory oversight in slaughtering practices, particularly the absence of meat inspection and supervision in abattoirs, rural areas, and small towns. Additionally, the improper disposal of offal and infected organs heightens disease transmission risks, perpetuating the infection cycle among livestock [44]. Comparatively, the frequency of CE in this study was lower than in regions such as Moldova (59.3%) [45] and Ethiopia (20.5%) [46], and recent trends indicate a decline in the CE incidence in Pakistan from 8.71% in 2018 to 6.22% in 2022 [48].

The findings of this study also reveal substantial knowledge and practice gaps among the farmers concerning improved animal care, zoonotic disease transmission, and effective dog management (Table S3). Notably, 66.5% of the farmers expressed interest in training for enhanced animal care practices; however, the awareness of zoonotic diseases associated with canine infections remains low, with only 20.5% recognizing the transmission risks from dogs to livestock. This disparity highlights an urgent need for educational initiatives to raise awareness about risks related to livestock-dog interactions.

The variety of livestock owned by the respondents, including buffaloes (39%), cows (23%), sheep (18.5%), and goats (12%), illustrates a complex farming ecosystem. While 51% of these animals grazed naturally, only 25% relied on streams for water. Although an 80.5% satisfaction rate regarding environmental cleanliness was reported, it may be misleading due to the inadequate dog management practices that undermine hygiene.

Dog ownership is prevalent, with 85% of households possessing dogs, and alarmingly, 94.08% of these dogs had unrestricted access to livestock. This presents significant potential health risks, as the primary motivations for dog ownership include guarding livestock, companionship, and herding. The management practices concerning dogs are concerning; 100% of the farmers admitted to feeding their dogs uncooked animal parts, which can compromise both canine and livestock health.

The urgency of health management practices is further emphasized by the fact that 70.41% of the farmers did not vaccinate their dogs. Given the direct contact between dogs and livestock, this lack of vaccination heightens the risk of zoonotic disease transmission, underscoring the necessity of educating the farmers on responsible pet ownership and veterinary care. Additionally, the practice of home slaughter during Eid ul-Adha, with 81% of the farmers opting for this method due to facility limitations, exacerbates hygiene issues, particularly as 53% disposed of animal offal on-site, contributing to environmental safety concerns.

In summary, while the interest in training among the farmers is high, there remains a critical need for comprehensive educational programs focused on animal care, responsible dog management, and hygiene practices. By addressing these knowledge gaps and enhancing the awareness of zoonotic risks, better livestock health outcomes and a safer agricultural environment can be achieved. Developing community-based training that incorporates effective veterinary practices, waste management, and hygiene protocols could significantly mitigate the identified risks. The economic impact of CE extends beyond the immediate loss of edible tissue; future research should quantify the proportion of organs that are condemned specifically due to CE lesions to better assess the full scope of the economic impacts on livestock owners. The findings underscore the importance of a holistic approach to livestock management that integrates animal health with public health considerations. On the other hand, as the current CE control in the study region relies on dog deworming and improved slaughter hygiene, the vaccination of livestock against *E. granulosus* could offer a complementary strategy. The EG95 vaccine, which confers immunity to sheep and other intermediate hosts by targeting the parasite’s oncosphere, has demonstrated >95% efficacy in trials and is used in several countries in Africa and Asia [67]. However, its adoption in Pakistan faces barriers, such as limited veterinary infrastructure, high costs, and low farmer awareness. For vaccination to become feasible, targeted subsidies, community education, and integration with existing livestock programs (e.g., brucellosis vaccination) would be essential. Additionally, vaccine deployment must align with One Health frameworks, combining livestock immunization with dog deworming and offal management to disrupt the parasite’s lifecycle. While not yet viable in resource-limited settings like Malakand, advancements in thermostable or oral vaccines could enhance accessibility in the future.

Lastly, while this study provides critical insights into the epidemiology and socioecological drivers of CE in Khyber Pakhtunkhwa, certain limitations must be acknowledged. First, abattoir-derived data inherently reflect non-random sampling, as animals are selected for slaughter based on market demand, health status, or productivity, potentially over-representing older or less productive livestock. Second, diagnostic reliance on visual inspection and palpation, while pragmatic in resource-limited settings, precludes the molecular confirmation of cyst viability or strain identification: a limitation well documented in similar field-based studies [39,53]. Third, the use of summary Chi-square tests, though appropriate for an exploratory analysis, may oversimplify multifactorial risk associations, and logistical constraints limit the application of regression models. Finally, the partial overlap between the abattoir and questionnaire respondents underscores the need for future work to integrate slaughterhouse surveillance with longitudinal farm-level monitoring.

Despite these constraints, the current findings establish a foundational benchmark for CE control in Pakistan and highlight actionable pathways for intervention, such as dog deworming and farmer education. To advance this work, we recommend prospective studies incorporating molecular diagnostics, larger cohorts, and community-based designs to disentangle confounders and quantify the economic burden of organ condemnation. Such efforts, aligned with One Health principles, could transform regional CE management while contributing to global zoonotic disease mitigation.

## 5. Conclusions

This study evaluated the frequency and associated risk factors of CE in livestock populations across five sub-regions of Malakand District, Pakistan: Batkhela, Bajawar, Chakdara, Dargai, and Thana. The study identified several factors, including sex, age, and animal species, that contribute to the prevalence of CE. However, it is important to note that a larger and more diverse sample would be necessary to thoroughly evaluate these factors. The transmission of CE is exacerbated by several critical factors, including close interactions between dogs and livestock in grazing areas, inadequate health education, unrestricted dog access to potentially infected offal, and poor waste management practices. Given the ongoing high incidence of CE, a One Health approach is strongly recommended to mitigate transmission risks by addressing inter-related animal, human, and environmental factors. Implementing effective control measures, such as stringent regulations on the disposal of infected offal and improving waste management systems, is essential, particularly in developing countries like Pakistan. Further research incorporating diverse animal hosts is necessary to enhance our understanding of CE’s eco-epidemiology and improve surveillance and preventive strategies in the region.

## Figures and Tables

**Table 1:** Prevalence of Cystic Echinococcosis (CE) in slaughtered livestock across species in Khyber Pakhtunkhwa, Pakistan (May 2022–February 2024).

Hosts	Total Examined Animals	Infected Animals	Frequency %
Buffaloes	455	30	6.59
Cattle	295	17	5.76
Sheep	200	11	5.5
Goats	170	5	2.94
Total	1120	63	5.62

**Table 2:** Region-specific prevalence of Cystic Echinococcosis (CE) in buffaloes, cows, goats, and sheep across district of Malakand, Pakistan.

Regions	Total Animals	Buffaloes	Cows	Sheep	Goats	Total Infected	Frequency %
Bajawar	300	110	80	60	50	18	6%
Batkhela	220	90	60	40	30	12	5.45%
Dargai	200	90	50	30	30	11	5.5%
Thana	200	85	55	30	30	12	6%
Chakdara	200	80	50	40	30	10	5%
Total	1120	455	295	200	170	63	5.62%
Chi^2^							32.47 **

Note: **, 0.01 significance.

**Table 3:** Organ-specific distribution of Cystic Echinococcosis (CE) lesions in buffaloes, cows, sheep, and goats in Khyber Pakhtunkhwa, Pakistan.

Organs	Liver I(t)/N (%)	Lungs I(t)/N (%)	Kidneys	Spleen	Heart	Total
Buffaloes	18/30 (60.0%)	10/30 (33.3%)	1/30 (3.3%)	0	1/30 (3.3%)	30
Cattle	9/17 (52.9%)	7/17 (41.1%)	1/17 (5.8%)	0	0	17
Sheep	5/11 (45.45%)	6/11 (54.5%)	0	0	0	11
Goats	2/5 (40%)	2/5 (40%)	0	0	1/5 (20.0%)	5
Total	34/63 (53.9%)	25/63 (39.6%)	2/63 (3.17%)	0	2/63 (3.17%)	63
Chi^2^	1.53 ^ns^	3.08 ^ns^	14.18 **	-	18.21 **	

Note: I(t), infected animals; N, total observed animals; (%), frequency percentage; ns; non-significant; **, 0.01 significance.

**Table 4:** Age-related prevalence of Cystic Echinococcosis (CE) in buffaloes, cows, sheep, and goats in Khyber Pakhtunkhwa, Pakistan.

Age	Buffaloes I(t)/N(%)	Cattle I(t)/N(%)	Sheep I(t)/N(%)	Goats I(t)/N(%)	Total I(t)/N(%)
1–3 Y	7/170 (4.11%)	3/106 (2.83%)	2/47 (4.25%)	1/57 (1.75%)	13/380 (3.42%)
3–5 Y	7/110 (6.36%)	5/71 (7.04%)	3/72 (4.16%)	1/41 (2.43%)	16/294 (5.44%)
5+ Y	16/175 (9.14%)	9/118 (7.62%)	6/81 (7.40%)	3/72 (4.16%)	34/446 (7.62%)
Total	30/455 (6.59%)	17/295 (5.76%)	11/200 (5.5%)	5/170 (2.94%)	63/1120 (5.62%)
Chi^2^	14.86 **	4.74 **	6.94 *	6.77 *	---

Note: I(t), infected animals; N, total observed animals; (%), frequency percentage; *, 0.05 significance; **, 0.01 significance.

**Table 5:** Sex-specific prevalence of Cystic Echinococcosis (CE) in buffaloes, cows, sheep, and goats in Khyber Pakhtunkhwa, Pakistan.

Livestock	Male I(t)/N(%)	Female I(t)/N(%)
Buffaloes	12/210 (5.71%)	18/245 (7.34%)
Cattle	5/113 (4.42%)	12/182 (6.59%)
Sheep	3/72 (4.16%)	7/128 (5.46%)
Goats	2/69 (2.89%)	4/101 (3.96%)
Total	22/464 (4.74%)	41/656 (6.25%)
Chi^2^	57.81 **	43.72 **

Note: I(t), infected animals; N, total observed animals; (%), frequency percentage; **, 0.01 significance.

## Data Availability

All data needed to evaluate the conclusions in this paper are present either in the main text or the Supplementary Materials.

## Supplementary Materials

The following supporting information can be downloaded at: https://www.mdpi.com/article/10.3390/ani15111617/s1. All data generated or analyzed during this study are included in this manuscript and its Supplementary Files; 1. Supplementary File S1—Supplementary Figures: Figure S1. The map of Pakistan that highlights the study areas within the Malakand District, specifically BATKHELA, BAJAWAR, CHAKDARA, DARGAI, and THANA. (A) The inset map illustrates the geographical context of Malakand within Pakistan (depicted in green) and Batkhela (shown in cyan). (B) The locations of the abattoirs are marked where the examined animals were sourced: (1) BATKHELA (light green), (2) BAJAWAR (cyan), (3) CHAKDARA (blue), (4) DARGAI (pink), and (5) THANA (yellow). Map Creation Details: Software Used: ArcGIS Pro (Version 3.x). 2. Supplementary File S2—Supplementary Tables: Table S1. Demographic and Socioeconomic Profile of Survey Respondents. Table S2. Livestock Management Practices, Health Measures, Education and Awareness of Zoonotic Diseases among Farmers. Table S3. Evaluation of Farmers’ Knowledge, Dog Ownership, and Practices Affecting Cystic Echinococcosis (CE) Transmission. Table S4. Livestock Slaughtered per Abattoir and Species in Malakand District, Pakistan. Table S5. Organ-Specific Cyst Burden and severity in studied livestock.
