# Supplementary material for: Investigating the Epidemiology and Socioecological Dynamics of Hydatid-like Cysts Within a Specific Endemic District
Source: Animals (Basel). 2025 May 30;15(11):1617. doi: 10.3390/ani15111617 (PMC12153600; doi:10.3390/ani15111617)
Supplement: Supplementary file 1 [file animals-15-01617-s001.zip › 1. Supplementary File S1—Supplementary Figures.pdf]

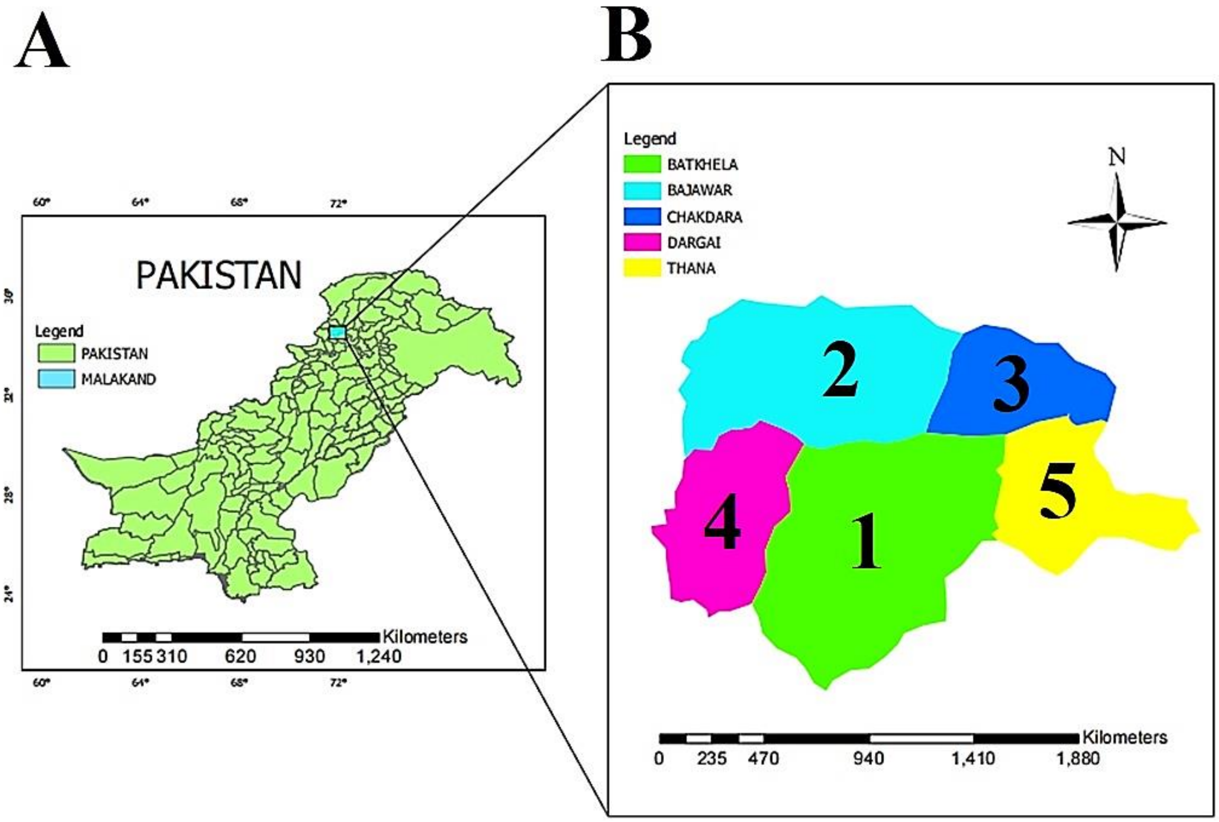

Figure S1. The map of Pakistan that highlights the study areas within the Malakand District, specifically BATKHELA, BAJAWAR, CHAKDARA, DARGAI, and THANA. **A)** The inset map illustrates the geographical context of Malakand within Pakistan (depicted in green) and Batkhela (shown in cyan). **B)** The locations of the abattoirs are marked where the examined animals were sourced**: 1)** BATKHELA (light green), **2)** BAJAWAR (cyan), **3)** CHAKDARA (blue), **4)** DARGAI (pink), and **5)** THANA (yellow). Map Creation Details: Software Used: ArcGIS Pro (Version 3.x).
