# Supplementary material for: Investigating the Epidemiology and Socioecological Dynamics of Hydatid-like Cysts Within a Specific Endemic District
Source: Animals (Basel). 2025 May 30;15(11):1617. doi: 10.3390/ani15111617 (PMC12153600; doi:10.3390/ani15111617)
Supplement: Supplementary file 1 [file animals-15-01617-s001.zip › 2. Supplementary File S2—Supplementary Tables.pdf]

Table S1. Demographic and Socioeconomic Profile of Survey Respondents.

S. N	Variables	Categories	Number	%
1	Regions	(1) Dargai (District Malakand)	31	15.5
		(2) Batkhela (District Malakand)	64	32
		(3) Thana (District Malakand)	29	14.5
		(4) Chakdara (District Lower Dir)	40	20
		(5) District Bajawar	36	18
2	Gender of the Farmer	(1) Male	187	93.5
		(2) Female	13	6.5
3	Age of the farmer	(1) 22-28	27	13.5
		(2) 29-34	42	21
		(3) 35-40	57	28.5
		(4) 40 above	74	37
4	Farmer position in the household	(1) Head of the House	147	73.5
		(2) Dependent	53	26.5
5	Residential Area of the Farmer	(1) Urban	64	32
		(2) Rural	136	68
6	Are you a migrant or a refugee	(1) Yes	31	15.5
		(3) No	169	84.5
7	Education of the farmer	(1) Primary	65	32.5
		(2) Middle	35	17.5
		(3) Matriculation & above	15	7.5
		(4) Religious education	12	6
		(5) Illiterate	73	36.5
8	Source of income	(1) Livestock farming	91	45.5
		(2) Crop farming	68	34
		(3) Other	41	20.5
9	Experience with livestock	(1) 1-4	12	6
		(2) 5-9	54	27
		(3) 10-14	79	39.5
		(4) 15-19	44	22
		(5) 20 and above	11	5.5

Table S2. Livestock Management Practices, Health Measures, Education and Awareness of Zoonotic Diseases among Farmers.

S. N	Variables	Categories	No	%
1	Animals Housing	(1) Inside House	132	66
		(2) Outside House	68	34
2	Types of Animals Raised:	(1) Buffalo	78	39
		(2) Cows	46	23
		(3) Sheep	37	18.5
		(4) Goats	24	12
		(5) More than one species	15	7.5
3	How often do you feed your animals per day?	(1) Once	12	6
		(2) Twice	121	60.5
		(3) Thrice or more	67	33.5
4	What type of feed do you provide to your animals?	(1) Natural grazing	102	51
		(2) Purchased feed	48	24
		(3) Homemade feed mix	29	14.5
		(4) Others	21	10.5
5	Do you provide supplements or vitamins to your animals?	(1) Yes	71	35.5
		(2) No	129	64.5
6	How often do you provide fresh water for your animals?	(1) Daily	113	56.5
		(2) Twice daily	43	21.5
		(3) Thrice daily	27	13.5
		(4) Other	18	9
7	What is the facility's water source?	(1) Municipal	21	10.5
		(2) Well	133	66.5
		(3) Stream	29	14.5
		(4) Rainwater	5	2.5
		(5) River	12	6
8	How often do you clean animal housing or pens?	(1) Daily	161	80.5
		(2) Weekly	30	15
		(3) Monthly	9	4.5
9	Do you monitor and manage animal pregnancies?	(1) Yes	141	70.5
		(2) No	39	19.5
		(3) Sometimes	20	10

S. N	Variables	Categories	No	%
10	Do you vaccinate your animals regularly?	(1) Monthly	26	13
		(2) Quarterly	51	25.5
		(3) Yearly	92	46
		(4) No	31	15.5
11	Are you aware of any government or veterinary programs to support animal care?	(1) Yes	177	88.5
		(2) No	23	11.5
12	Would you be interested in receiving training or information about improved animal care practices?	(1) Yes	131	66.5
		(2) No	69	34.5
13	Have you observed any common diseases or health issues in your animals?	(1) Yes	189	94.5
		(2) No	11	5.5
14	Are you aware of the disease cystic echinococcosis (CE)?	(1) Yes	41	20.5
		(2) No	149	74.5
15	If yes, then do you know that the Echinococcus parasite causes CE?	(1) Yes	27	65.85
		(2) No	14	34.14
16	If yes, then do you know about the signs, symptoms, and risks of CE?	(1) Yes	20	74.07
		(2) No	7	25.92
17	Do you take any preventive measures to control parasites in your animals?	(1) Yes	29	14.5
		(2) No	171	85.5
18	If you are aware of CE, please describe any signs, symptoms, or risks you are familiar with:	Open-Ended Question		

Table S3. Evaluation of Farmers' Knowledge, Dog Ownership, and Practices Affecting Cystic Echinococcosis (CE) Transmission

S.N.	Variable	Categories	No	%
1	Do you know how CE affects animal organs, specifically the liver and lungs?	(1) Yes (2) No	200	100.0
2	Do you know that CE can be transmitted to humans?	(1) Yes (2) No	41	20.5
		(2) No	159	79.5
3	If yes, then how would you rate your knowledge of echinococcosis?	(1) Good (2) Moderate (3) Limited	15	36.58
		(2) Moderate	12	29.26
		(3) Limited	14	34.14
4	Have you heard or met anyone diagnosed with CE at any hospital in the village/household?	(1) Yes (2) No	79	39.5
		(2) No	121	60.5
5	Do you know that dogs can act as hosts for the parasite causing CE?	(1) Yes (2) No	133	66.5
		(2) No	67	33.5
6	Do you currently keep any dogs in or around your household or farm?	(1) Yes (2) No	170	85.0
		(2) No	30	15.0
7	If yes, how many dogs do you keep?	(1) 1 (2) 2 (3) More than 2	113	66.86
		(2) 2	37	21.89
		(3) More than 2	19	11.24
8	What is the primary reason for keeping dogs on the property?	(1) Guarding livestock (2) Companionship	96	56.8
		(3) Herding Animals (4) Other	41	24.26
			20	11.8
			12	7.10
9	Where do your dogs primarily stay?	(1) Inside the house (2) Inside the farm area	65	38.46
		(3) Separate shelter	68	42.23
			29	17.15
10	Are your dogs allowed to roam freely with livestock?	(1) Yes (2) No	159	94.08
		(2) No	10	5.91
11	How often do your dogs come into direct contact with livestock?	(1) Daily (2) Rarely (3) Never	160	94.67
		(2) Rarely	9	5.32
		(3) Never	0	0.00
12	Are your dogs vaccinated regularly?	(1) Yes (2) No	50	29.58
		(2) No	119	70.41
13	Do you believe that dogs can transmit diseases to livestock?	(1) Yes (2) No	59	29.5

S.N.	Variable	Categories	No	%
		(2) No	141	70.5
14	Have you received any information on how to prevent diseases that could spread from dogs to livestock?	(1) Yes (2) No	190	95.0
		(2) No	10	5.0
15	If yes, how do you prevent disease transmission from dogs to livestock?	(1) Regular vet check-ups (2) Proper disposal	19	9.5
		(2) Proper disposal of dog waste	141	70.5
		(3) Separate living areas (4) Other	27	13.5
			13	6.5
16	Do you feed your dogs any raw meat or livestock by-products?	(1) Yes (2) No	193	96.5
		(2) No	7	3.5
17	Do your dog(s) consume offal?	(1) Yes (2) No	200	100.0
		(2) No	0	0.0
18	If yes, how is the offal prepared?	(1) Raw (2) Fried (3) Roasted (4) Boiled	200	100.0
			0	0.0
			0	0.0
			0	0.0
19	Have you slaughtered any livestock at home in the last 12 months?	(1) Yes (2) No	114	57.0
		(2) No	86	43.0
20	Where did you perform your slaughter during Eidul Adha?	(1) Slaughterhouse (2) House	19	9.5
		(3) Nearby (4) Other	162	81.0
			13	6.5
			6	3.0
21	What do you do with the offal of animals when you perform your slaughter during Eidul Adha?	(1) Bury (2) Burn	33	16.5
		(3) Open Area (4) Throw into Rivers	18	9.0
			106	53.0
			43	21.5
22	Is there a slaughterhouse nearby?	(1) Yes (2) No	67	33.5
		(2) No	133	66.5
23	What do you do with livestock that die on their own?	(1) Bury (2) Burn	91	45.5
		(3) Open Area (4) Throw into Rivers	24	12.0
			39	19.5
			46	23.0

S.N.	Variable	Categories	No	%
24	Do your dogs go out to pasture with the cattle when the animals are being herded?	(1) Yes (2) No (3) Sometimes	69	34.5
			119	59.5
			12	6.0
25	Do your animals graze areas where dogs defecate?	(1) Yes (2) No	93	46.5
		(2) No	107	53.5
26	Are your dogs ever treated by veterinary staff when they are sick?	(1) Yes (2) No	11	5.5
		(2) No	189	94.5
27	Have your dogs ever been de-wormed?	(1) Yes (2) No	19	9.5
		(2) No	181	90.5

Table S4. Livestock Slaughtered per Abattoir and Species in Malakand District, Pakistan

Slaughterhouse Location	Buffaloes	Cows	Sheep	Goats	Total
Batkhela 1	45	30	20	15	110
Batkhela 2	45	30	20	15	110
Thana 1	42	28	15	15	100
Thana 2	43	27	15	15	100
Dargai 1	40	25	15	10	90
Dargai 2	40	25	15	10	90
Bajawar 1	55	40	25	20	140
Bajawar 2	55	40	25	20	140
Chakdara 1	40	25	20	15	100
Chakdara 2	40	25	20	15	100
Total	455	295	200	170	1,120

Table S5. Organ-Specific Cyst Burden and severity in studied livestock

Organ	Total Cysts	Minor (%)	Intermediate (%)	Major (%)
Liver	124	15 (53.6%)	8 (36.4%)	11 (84.6%)
Lungs	76	10 (35.7%)	12 (54.5%)	2 (15.4%)
Kidneys	10	3 (10.7%)	2 (9.1%)	0 (0%)
Heart	4	0 (0%)	0 (0%)	0 (0%)
**Total**	**214**	**28**	**22**	**13**
